# Effects of communication training with the MAAS-Global-D instrument on the antibiotic prescribing for respiratory infections in primary care: study protocol of a randomised controlled trial

**DOI:** 10.1186/s13063-016-1293-5

**Published:** 2016-04-02

**Authors:** Friederike Hammersen, Katja Goetz, Andreas Soennichsen, Timo Emcke, Jost Steinhaeuser

**Affiliations:** Institute of Family Medicine, University Hospital of Luebeck (UKSH), Campus Luebeck, Ratzeburger Allee 160, 23562 Luebeck, Germany; Institute of General Practice and Family Medicine, Faculty of Health, Witten/Herdecke University, Alfred-Herrhausen-Str. 50, 58448 Witten, Germany; Association of Statutory Health Insurance Physicians Schleswig-Holstein, Bismarckallee 1-6, 23795 Bad Segeberg, Germany

**Keywords:** MAAS-Global-D, Communication skills, Physician-patient relationship, Antibiotic prescribing, Primary health care, Evidence-based medicine, Guidelines, Communicative competencies, Routine data, Respiratory tract infections

## Abstract

**Background:**

Primary care physicians account for the majority of antibiotic prescribing in ambulatory care in Germany. Respiratory diseases are, regardless of effectiveness, often treated with antibiotics. Research has found this use without indication to be caused largely by communication problems (e.g. expectations on the patient’s part or false assumptions about them by the physician). The present randomised controlled trial (RCT) study evaluates whether communication training for primary care physicians can reduce the antibiotic prescribing rate for respiratory tract infections.

**Methods/Design:**

The study consists of three groups: group A will receive communication training; group B will be given the same, plus additional, access to an evidence-based point-of-care tool; and group C will function as the control group. The primary endpoint is the difference between intervention and control groups regarding the antibiotic prescribing rate before and after the intervention assessed through routine data. The communication skills are captured with the help of the communication instrument MAAS-Global-D, as well as individual videos of physician-patient consultations recorded by the primary care physicians. These skills will also be regarded with respect to the antibiotic prescribing rate.

A process evaluation using qualitative as well as quantitative methods should provide information about barriers and enablers to implementing the communication training.

**Discussion:**

The trial contributes to an insight into the effectiveness of the different components to reduce antibiotic prescribing, which will also be supported by an extensive evaluation. Communication training could be an effective method of reducing antibiotic prescribing in primary care.

**Trial registration:**

DRKS00009566

**Date registration:**

5 November 2015.

## Background

Primary care physicians account for 67 % of antibiotic prescribing in ambulatory care in Germany [1]. In their family practices, respiratory diseases rank among the most frequent reasons for consulting [2]. In many of these cases antibiotic prescribing is not indicated and guidelines for bronchitis, sinusitis and pharyngitis recommend abstaining from antibiotic treatment for the vast majority of cases [3–7]. Rates of approximately 31 % antibiotic prescribing for respiratory diseases and approximately 60 % for pharyngitis/tonsillitis, however, leave room for substantial improvement [8]. Unnecessary use of antibiotics causes not only adverse drug reactions, but also contributes to antibiotic resistance and unnecessary health expenditure [9, 10]. It also reinforces patients’ incorrect assumptions regarding the effectiveness of antibiotics for respiratory diseases, which in turn influences their expectations when suffering from similar symptoms later on and triggers their belief in the need for antibiotics [11, 12]. Studies have found that inappropriate prescriptions of antibiotics cannot be attributed solely to a lack of knowledge in primary care physicians (PCPs). Equally important are falsely assumed patient expectations with regard to receiving medication [11, 13] in addition to PCPs feeling pressured by patients who ask for such medication [14] and wishing to maintain good relationships with patients or to avoid confrontations and the need to explain the differences between viruses and bacteria [11]. Physicians state that explaining takes up time and that explanations are often not understood by the patient, whereas prescribing antibiotics is a quicker and simpler course of action [11].

Studies have revealed the possibility of decreasing antibiotic prescribing in primary care by enhancing PCPs’ communication skills [15–17]. Communication training proved to be an effective intervention, sometimes paired with further elements, such as point-of-care testing, delayed prescribing or additional patient education material [12, 16–19]. The achieved effects were an absolute reduction of 10–12 % in the rate of antibiotic prescribing [15–17], corresponding to a relative reduction of 25-40 % [16, 18]. This reduction of antibiotic prescribing does not, or only to a very modest extent, impact negatively on patient satisfaction [12, 16, 17, 19]. Furthermore, a review on interventions to improve antibiotic prescribing concluded that multiple interventions, which contain more than one interventional module, are more likely to be effective than single intervention strategies [15]. Of the tested combinations of modules, educational meetings with educational material for the physician scored best [15]. Additionally, active clinician education strategies (e.g. interactive workshops) appear to be superior to passive ones (e.g. educational material only) [16].

Adequate communication skills are probably more important in primary care than in any other medical specialty. However, the German specialist medical training for PCPs lacks nationwide, structured training on communication skills [20]. This is in contrast to the situation in the Netherlands, for example, where an instrument to measure physicians’ communication and medical skills is incorporated in a structured curriculum on communication skills. The physicians record videos of physician-patient consultations which are then rated using the instrument produced to examine these skills. The instrument is now widely used in medical schools as well as in specialist training [21]. This instrument, named MAAS-Global-D, was recently translated and adapted for use in Germany [22]. Against this background, our study aims to investigate whether the rate of antibiotic prescribing can be reduced with the help of MAAS-Global-D-based communication training. We postulated the following research hypothesis:

In the intervention groups, the rate of antibiotic prescribing by PCPs for acute bronchitis, sinusitis and pharyngitis decreases following the communication training compared to the control group with no intervention.

Based on the MAAS-Global-D, we designed a multiple-component intervention, which, in brief, includes:

- Communication training for PCPs with interactive elements: the programme will be suitable for PCPs with professional experience. After the training sessions, the PCPs submit videos displaying their own communication with patients and receive feedback on it.
- Further information material for PCPs: they will be educated in the usage of Evidence-based Medicine – Guidelines (EbMG), a point-of-care tool, which will be made available online to one of the intervention groups during the whole study period and which aims to support and facilitate decision-making [23].

The groups compared will be:

Group A: Intervention group A with communication training
Group B: Intervention group B, same training as group A plus access to EbMG
Group C: Control group C with no intervention

By assessing and evaluating the communication training, it can be adapted and possibly also improved. Since the MAAS-Global-D instrument, as well as the associated manual, are published for free download, they will be available in order to establish nationwide training to decrease antibiotic prescribing in primary care and could be implemented in specialty training for PCPs later on.

### Objectives

#### Primary objective

To assess whether communication training for PCPs working in family practices in the federal state of Schleswig-Holstein (SH) in northern Germany leads to a reduction of antibiotic prescribing rates for acute bronchitis, sinusitis and pharyngitis compared to a control group (A + B versus C).

### Secondary objectives

1. To assess whether provision and training in the usage of EbMG leads to an additional reduction of antibiotic prescribing (A versus B)
2. To assess whether PCPs who score higher on the MAAS-Global-D instrument (meaning they have better communication skills) as measured through videos of their consultations prescribe antibiotics less often than PCPs with a lower score
3. To evaluate the communication training as well as the use and the usefulness of EbMG by interviewing the PCPs in a process evaluation

## Methods/Design

### Trial design

The trial is designed as a randomised, controlled trial postulating superiority of the interventions over conventional care in family practices. The study consists of three arms and a primary endpoint of antibiotic prescribing rates of PCPs for acute bronchitis, sinusitis and pharyngitis over a 3-month period starting around 1 month after the communication training and education in the usage of EbMG. Randomisation of either of the two intervention groups or the control group will be performed at practice level with a 1:1:1 allocation. Therefore, random permuted blocks will be generated. Due to the data format, practices with more than one physician represent one case. The person assigning the participants to the groups will do so blinded. Further blinding does not fit the trial design.

### Study setting

The trial will be conducted in the population of PCPs in SH. In total, there are about 1900 PCPs in SH who are working in family practices, including rural as well as urban sites. In Germany, all resident PCPs belong to a subdivision of the Association of Statutory Health Insurance Physicians (KV). These subdivisions are responsible for the remuneration of physicians’ services when treating patients who are covered by statutory health insurance – approximately 87 % of all inhabitants [24]. Since 2011, all practices belonging to KV are obligated to do their accounting online, which requires a connection to the data centre of their KV. These routine data of the KV of Schleswig-Holstein (KVSH) will be the data basis for the present study concerning data about diagnoses. Information about prescriptions are obtained through a more complex process including pharmacies and the Central Research Institute of Ambulatory Health Care in Germany (ZI), but will also be provided by a federal subdivision of the KV together with data about diagnoses. Therefore, all data is referred to as ‘routine data’ in this article. For our objectives it is necessary to establish a connection between the PCPs and their data on diagnoses and antibiotic prescriptions. Each practice possesses an identification number, which can be used for this purpose. The study centre is located at the Institute of Family Medicine at the University Hospital of Luebeck (UKSH), SH. It will work in close collaboration with a KVSH data manager for the purpose of data analysis. Prior to analysis, official approval from the ‘Independent Regional Centre for Data Protection’ (Unabhaengiges Landeszentrum fuer Datenschutz) of the federal state of SH is sought.

### Interventions

The study incorporates a multiple-component intervention corresponding to the two interventional study arms (group A and B). Both intervention groups receive communication training with an interactive workshop character (4.5 hours of input), which is held at the Institute of Family Medicine. It is designed as a face-to-face delivery by members of the research team, including an expert on physician-patient communication.

Firstly, the speakers establish the relevance and success of physician-patient communication. The speakers then provide input concerning the associated evidence base. Furthermore, the participants learn the different communicative phases of a consultation, corresponding communication skills as well as general communication skills for the whole consultation (e.g. adequate provision of information, structuring and empathy, shared decision-making). These topics also function as an introduction to the MAAS-Global-D rating scale. The PCPs have the opportunity to evaluate a video with the recent learned criteria of the MAAS-Global-D, as well as to perform a consultation with a patient-actor themselves. The curriculum is derived from the aforementioned structured Dutch communication training, the MAAS-Global-D instrument, as well as from further evidence about successful physician-patient communication.

Group B receives an additional 1.5 hours of face-to-face input comprising education in the use of EbMG online. With online-access to this point-of-care tool, further information material on the prescribing of antibiotics for uncomplicated respiratory infections is provided for group B. The German version of the EbMG, which was originally developed in Finland, incorporates a regularly updated point-of-care tool with guidelines based on current best evidence, including videos, audio samples, pictures, links to evidence summaries and articles from the Cochrane library in addition to ready-to-print patient information [23]. It has received National Institute for Health Clinical Excellence (NICE) accreditation [25].

Another part of the intervention for both groups targets organisational matters: video recording in the PCPs’ practices, how to install the webcam and the informed consent forms for all patients. Each PCP receives a secure USB stick with a password that is used to transfer the videos to the study centre. To increase adherence, a monthly email will be sent to the participants reminding them of the videos and the book vouchers for each video.

The control group does not receive any intervention and provides ‘usual’ care to patients.

### Eligibility criteria

Written informed consent will thus be obtained from all participants: PCPs, their colleagues as well as patients to be filmed.

### PCPs

A PCP is defined as either a general practitioner or an internist working in primary care. Inclusion criteria are:

- PCPs who have been active members of KVSH for a minimum of 5 years
- provide written, informed consent
- work as a PCP in a practice, and
- in case they work with several PCPs in a group practice, all of them must give written, informed consent, since it is expected that a PCP in this working environment who receives the communication training will influence his colleagues

The exclusion criterion is:

- withdrawal of informed consent

### Patients

The patients involved in the video recording are recruited by the PCPs themselves in their own practices. Inclusion criteria are:

- written, informed consent before recording
- minimum age of 18 years
- a first consultation about acute symptoms (though not necessarily about respiratory infections)^1^

The exclusion criterion is:

- insufficient knowledge of the German language

### Outcomes

#### Primary outcome measures

- Evaluation of the antibiotic prescribing rate in both intervention groups compared to the control group between baseline (meaning the prescribing rate of the three periods April–June 2013, 2014, and 2015) and post-intervention (April–June 2016)

To determine the antibiotic prescribing rate for each PCP, all patients with the target-diagnoses of acute bronchitis, sinusitis and pharyngitis (classified by the *International Classification of Diseases*, *version 10* (ICD-10) codes: J01.-; J02.-; J20.- [26]) are identified in the routine data of KVSH and the proportion of these patients who receive antibiotic prescriptions is calculated. The three diagnoses and corresponding antibiotic prescriptions are condensed into one rate for each PCP (in %). This will be completed for two time periods (*t*_0_ = baseline, before intervention: mean of quarter II in the years 2013–2015; *t*_1_ = post-intervention: quarter II in the year 2016). For each PCP the change from *t*_0_ to *t*_1_ will be calculated (rate *t*_1_-rate *t*_0_). For each study group, the mean of the antibiotic prescribing rates of all PCPs in the group (at *t*_0_ and *t*_1_) will be calculated. For our primary endpoint the two intervention groups will be regarded as one group which is compared to group C.

#### Inclusion and exclusion criteria for data

To obtain the proportion of cases with the defined diagnoses and antibiotic prescribing for each PCP, the routine data are filtered for the following: inclusion of all cases with at least one visit to a PCP during time period *t*_0_ or *t*_1_, respectively, who were 18 years or older, had a diagnosis of acute bronchitis, sinusitis or pharyngitis. Exclusion if diagnosis of a further (bacterial) infection in the same quarter (ICD-10 codes A00.- till A37.-, A39.- till A79.-, J15.-, J17.- and J18.-). These cases need to be excluded because data about diagnoses are only available for each quarter and not on a daily basis. Furthermore, KVSH data about prescriptions cannot be linked directly to these diagnoses. If a patient is prescribed an antibiotic, it could either be for one of the defined diagnoses or another bacterial infection in the same quarter of the year. Hence all patients with the latter diagnoses are excluded. Furthermore, cases are excluded if multiple diagnoses of the defined diseases are made or the same diagnosis is made multiple times. Additionally, cases are excluded if they are diagnosed with a specific chronic disease (ICD-10 codes I50.-, J44.-, J45.-, C00-C75) or have a diagnosis related to pregnancy or childbirth (ICD-10 codes O). The presence of these diagnoses or the same diagnosis multiple times might qualify them as cases at higher risk or indicate a recurring disease where antibiotic prescribing is indicated. Eligible antibiotics are systemic antibiotics (40 different ATC codes, identified in [2]).

Using routine data is a fairly objective method in analysing prescribing behaviour compared to subjective methods such as interviewing patients retrospectively about their diagnosis and the antibiotics they received. Although this corresponds with high reliability, the validity might be compromised. ICD-10 codes and routine data might not represent the actual diagnoses and reasons for the consultation, but instead need to be completed for administrative reasons. Nevertheless, these limitations of routine data affect both intervention and control groups equally and, therefore, should not interfere with our results. Moreover, routine data is collected anyway and thus physicians are not burdened with additional documentation when participating in the study.

#### Secondary outcomes measures

- Evaluation of antibiotic prescribing rate in intervention group A versus intervention group B between *t*_0_ and *t*_1_

The antibiotic prescribing rates calculated for the primary outcome will also be used for the analysis of the secondary outcomes, i.e. the mean of change in the antibiotic prescribing rate from *t*_0_ to *t*_1_. Study groups A and B will be compared to assess whether there is an additional effect in study group B due to using EbMG.

- Evaluation of antibiotic prescribing rates depending on PCPs’ communication skills

PCPs’ communication skills will be defined over their MAAS-Global-D score, which is computed by rating the PCPs’ videos with the MAAS-Global-D instrument. For this only the PCPs of the intervention groups are considered. For each PCP a mean score is calculated based on the six videos recorded during *t*_1_.

To evaluate the association between the MAAS-Global-D score and the antibiotic prescribing rate during *t*_1_, a linear regression will be calculated with each PCP’s antibiotic prescribing rate at *t*_1_ as dependent variable. Independent variables to be included in the model are the MAAS-Global-D score, the antibiotic prescribing rate at *t*_0_ and the difference between the two rates, the PCP’s field of specialisation, their kind of practice (single versus shared) and its location (urban versus rural) as well as the number of patients per quarter.

- To evaluate the communication training as well as the use and the usefulness of the EbMG by interviewing the PCPs

This process evaluation will be undertaken after *t*_1_ in terms of a qualitative, guided interview. As it is unlikely that all PCPs shall be willing to participate in the qualitative interview, additionally every participant is asked to fill in a semi-structured questionnaire with open and closed questions. The topics of both interviews will concern whether and in what way the communication training was helpful, which elements of it the PCPs used, further enablers and barriers to implementing our communication recommendations as well as additional feedback about the communication training.

**Fig. 1:**
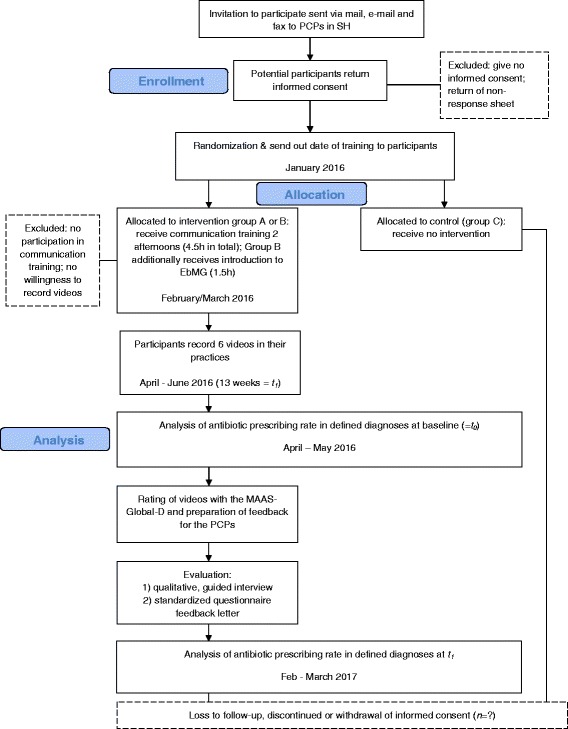
Flow diagram participant timeline. SH = Schleswig-Holstein, federal state in northern Germany; PCPs = primary care physicians; h = hours; EbMG = Evidence-based Medicine – Guidelines; *t*_0_ = baseline, pre-intervention time period; *t*_1_ = post-intervention time period; *n* = number of cases

A participant timeline along with the outcomes is provided in detail in the flow diagram in Fig. 1.

### Sample size

#### Primary outcome: evaluation of antibiotic prescribing rate in intervention groups compared to control group

Previous research has established that communication training can lead to a reduction of 12 % (95 % confidence interval −18.9 to −4.0) in antibiotic prescribing [17], with a mean of −4 % (standard deviation (SD) 15.6) in the intervention group and +8 % (SD 19.2) in the control group, respectively, from *t*_0_ to *t*_1_.

Additional definitions for sample size calculations are a power of 80 % and *α* = 5 %. A *t* test for unequal group sizes (Satterthwaite’s *t* test) results in 28 PCPs per study arm. Including an estimated dropout of 10 %, the total is 93 participants, yielding 31 per study arm.

### Recruitment of participants

All PCPs who have been members of the KVSH for at least the last five preceding years and who have made diagnoses of the defined ones are contacted via mail (*n* = 977). Furthermore, the information about the study and the invitation are spread through different channels: the Hausaerzteverband SH, which is a federal division of the biggest association of PCPs in Germany, further regional associations (Aerztenetze) as well as in the group of medical student trainers at the University of Luebeck.

Besides information on the study and the informed consent form, the invitation letter highlights the incentives for participants: credit points for continuing medical education as well as book vouchers. If more eligible PCPs apply for participation than needed, the first 93 are included and a waiting list is created.

### Data collection and management

#### Diagnoses and antibiotic prescribing

KVSH provides data about diagnoses to ZI over an intermediate body. ZI also has data on redeemed antibiotic prescriptions. Diagnoses and prescriptions are not originally linked but data sources are joined by ZI and provided as a joined data stream to KVSH. This routine data by KVSH forms the basis of a shortened dataset for the study team. Individual patients cannot be identified in the data set due to anonymisation.

#### Communication skills

During *t*_1_, PCPs in the intervention groups record six videos of consultations in their practice. The PCPs choose the videos themselves (preferably ones in which the PCP recognises their own usual behaviour) and send them via a secure USB stick to the study centre. The videos are then rated by researchers using the MAAS-Global-D rating scale. The English version of the instrument has been tested for reliability with Cronbach’s *α* and showed values of 0.78 - 0.86 [27, 28]. Furthermore, a comparison of the rating scale and the Common Ground instrument showed an acceptable value (0.59), which is an indicator for convergent validity [28].

### Data quality

A short questionnaire about the PCPs’ socio-demographics, the results of the MAAS-Global-D rating and the data from the process evaluation will be entered locally at the study centre. Where applicable, values can be chosen from a list of codes and their meaning is captured in a codebook. To secure and promote data quality, all data will be checked for double data entry and plausibility of range of values before analysis. Potential changes to the dataset are documented in a separate file.

### Analysis

#### Statistical methods concerning study outcomes

##### Primary outcome

All randomised PCPs will be included and analysed as randomised. The combined intervention groups’ rate (group A + B) will be compared to the control group (C) with a *t* test for the group mean (= proportion of antibiotic prescribing). As an effect size the difference of differences or difference of change [16], respectively, is chosen. This depicts the difference from *t*_0_ to *t*_1_ between the intervention and control groups.

##### Secondary outcomes

The additional effect of the EbMG will be evaluated similarly to the primary outcome under consideration of group A versus group B. Since the sample size is calculated with regard to the primary outcome, these analyses are only exploratory.

For further secondary outcomes, PCPs who adhered to the protocol will be encompassed in the analyses. Adherence refers to the intervention groups and whether each PCP has submitted a minimum of at least four videos. To explore differences in the antibiotic prescribing rate at *t*_1_ depending on the communication skills of the PCPs in group A and B, linear regression analyses will be conducted.

Quantitative data from the evaluation questionnaire will be presented descriptively only.

For all analyses, 95 % confidence intervals will be calculated where applicable.

### Additional analyses

Depending on the scale level and the distribution, parametric or non-parametric methods (e.g. Chi-squared test, *t* test or Mann-Whitney *U* test) will be applied to ascertain whether differences exist between intervention groups (A + B) and the control group at baseline.

### Ethics

PCPs and their filmed patients will participate voluntarily and may withdraw from participation at any time without giving reasons. No data monitoring committee is necessary because of the known minimal to negligible risks of the intervention. No adverse events are to be expected in this kind of study. However, participants (PCPs) and the filmed patients can contact the study team with further enquiries.

Due to the fact that prescribing data is pseudonymised by an external position of trust at ZI and the diagnoses are transferred in compliance with the legal requirements of the national data protectionist, no data can be reduced to a specific person.

Moreover, values which will be interpreted in the analyses are an aggregate function of pseudonymised prescription data in terms of the patient. The reference to the individual patient is no longer possible and this is in line with this part of data protection. The second and sufficient part of data protection, which refers to the misuse of routinely collected data in the research project, will be ensured with the help of a statutory application at the Independent Centre for Data Protection SH (ULD), which is in frame for such cases.

The study was approved by the ethics committee of Luebeck University before the recruitment of participants on 9 June 2015 (number of approval: 15-139).

## Discussion

The MAAS-Global-D instrument, which measures communicative and medical skills, has recently been translated into German [22] and will be tested in a randomised controlled trial in primary care in Germany for the first time. The trial design (three arms: multiple interventional elements and comparison with a control group) contributes to an insight into the effectiveness of the different components to reduce antibiotic prescribing, which will also be supported by extensive evaluation. Thereby, barriers and enablers to implement parts of or the whole of the communication training on a large scale will be worked out. The choice of active clinician education procedures and further educational material for the PCP, such as EbMG, would appear to be promising intervention strategies [15, 16].

### Strengths and limitations of the study

The randomisation will ensure a random distribution of characteristics and probably create nearly equal groups. Due to the number of participants, a prior stratification would not be reasonable. A limitation of the study is that randomisation must be carried out at practice level because the routine data cannot be assigned to the individual PCP in group practices. Therefore, untrained PCPs in group practices might bias the effect of the intervention.

Using the PCPs’ routine data instead of self-documentation bears the advantage that the data is not subject to selection bias: for example, if a PCP does not include all cases with the defined diagnoses in their documentation or if patients deny being in the study. Also, recall bias or effects due to the interviewer can be excluded. The Hawthorne effect is probably smaller, too, as the PCPs’ attention is not constantly drawn to patients with the relevant diagnoses. The routine data further allows the possibility of including several years as *t*_*0*_, which can balance extremes due to different seasonal variations. Extremes in *t*_1_ cannot be balanced, but a rise in antibiotic prescriptions due to a growth in infections in this time period would equally affect intervention and control groups.

Routine data is, at the same time, secondary data with known limitations: an often poorer data quality caused, for example, by misclassifications goes along with no possibility of external validation (e.g. of diagnoses) in our subsample. Further weaknesses are the lack of information on prescriptions that were not redeemed and using data covering only those patients who have statutory health insurance. Due to the data format, we do not have any further information about the ongoing of a patient’s disease, e.g. whether patients who did not use antibiotics suffer from complications afterwards. Information about side effects of antibiotics is also not available. However, these important issues will be part of the process evaluation.

Sample size calculation for this study was difficult because not only the effect size but also the *t*_0_ prescribing rate of antibiotics for the three diagnoses in Germany are based on estimates. Additionally, as in many trials, we will also be confronted with the self-selection bias of PCPs. Those interested or already sensitised for the issues (antibiotic prescription, communication skills) are more likely to participate in our study.

However, if our hypothesis is not rejected, the communication training is an easy and cost-effective way to reduce antibiotic prescribing for PCPs in Germany, who are facing symptoms of respiratory infections on a daily basis and need to make decisions about treatment.

### Trial status

The trial has been registered in the German Clinical Trial Register (DRKS00009566) and is currently recruiting participants

## Abbreviations

EbMG: Evidence-based Medicine – Guidelines
KV: Association of Statutory Health Insurance Physicians
KVSH: Association of Statutory Health Insurance Physicians Schleswig-Holstein
*t*_0_: baseline: pre-intervention time period (quarter II in years 2013–2015)
*t*_1_: post-intervention time period (quarter II in 2016)
PCP: primary care physician
SH: Schleswig-Holstein, federal state in northern Germany
ULD: Independent Centre for Data Protection SH (Unabhaengiges Landeszentrum fuer Datenschutz)
ZI: Central Research Institute of Ambulatory Health Care in Germany

## Footnotes

1 It is expected that PCPs can adopt the acquired basics concerning communication skills to different causes of consultations. In order to enable a rating of the videos, no follow-up meetings should be chosen because of lack of information regarding the prior consultation.
